# Crystal structures of murine and human Histamine-Releasing Factor (HRF/TCTP) and a model for HRF dimerisation in mast cell activation

**DOI:** 10.1016/j.molimm.2017.11.022

**Published:** 2018-01

**Authors:** Katy A. Doré, Jun-ichi Kashiwakura, James M. McDonnell, Hannah J. Gould, Toshiaki Kawakami, Brian J. Sutton, Anna M. Davies

**Affiliations:** a King’s College London, Randall Centre for Cell and Molecular Biophysics, New Hunt’s House, London, SE1 1UL, United Kingdom; b Medical Research Council & Asthma UK Centre in Allergic Mechanisms of Asthma, London, United Kingdom; c Laboratory for Allergic Disease, RIKEN Center for Integrative Medical Sciences, Yokohama, 230-0045, Japan; d Division of Cell Biology, La Jolla Institute for Allergy and Immunology, La Jolla, CA, 92037, USA; e Department of Dermatology, University of California San Diego, School of Medicine, La Jolla, CA, 92093, USA

**Keywords:** hHRF, human Histamin e-Releasing Factor, mHRF, murine Histamine-Releasing Factor, Immunoglobulin E, Allergy, Histamine-Releasing Factor

## Abstract

In allergic disease, mast cell activation is conventionally triggered by allergen-mediated cross-linking of receptor-bound IgE on the cell surface. In addition to its diverse range of intracellular roles in apoptosis, cell proliferation and cancer, Histamine-Releasing Factor (HRF) also activates mast cells and basophils. A subset of IgE antibodies bind HRF through their Fab regions, and two IgE binding sites on HRF have been mapped. HRF can form dimers, and a disulphide-linked dimer is critical for activity. The current model for the activity of HRF in mast cell activation involves cross-linking of receptor-bound IgE by dimeric HRF, mediated by HRF/Fab interactions. HRF crystal and solution structures have provided little insight into either the formation of disulphide-linked HRF dimers or the ability of HRF to activate mast cells. We report the first crystal structure of murine HRF (mHRF) to 4.0 Å resolution, revealing a conserved fold. We also solved the structure of human HRF (hHRF) in two new crystal forms, one at the highest resolution (1.4 Å) yet reported. The high resolution hHRF structure reveals a disulphide-linked dimer, in which the two molecules are closely associated, and provides a model for the role of both human and murine HRF in mast cell activation.

## Highlights

• First crystal structure of murine HRF solved.
• Crystal structure of human HRF solved at the highest resolution yet reported.
• Human HRF structure contains a disulphide-linked dimer.
• Dimer reveals a model for the role of human and murine HRF in mast cell activation.

## 1 Introduction

Histamine-Releasing Factor (HRF), also referred to as Translationally Controlled Tumour Protein (TCTP), p21, p23 and fortilin, is ubiquitously expressed in eukaryotes, and involved in apoptosis, cell cycle progression, cell proliferation and cancer (Bommer and Thiele, 2004, Nagano-Ito and Ichikawa, 2012, Bommer, 2012). In addition to its diverse range of intracellular roles, HRF is also able to activate mast cells and basophils, with the release of histamine (MacDonald et al., 1995, Kashiwakura et al., 2012), implicating a role in allergic disease. Mast cell activation is conventionally triggered by allergen-mediated cross-linking of FcεRI-bound IgE on the cell surface (Gould and Sutton, 2008). Studies of murine HRF (mHRF), which is 96% identical to human HRF (hHRF), revealed that a subset of IgE antibodies bound to mHRF through their Fab regions, and two IgE binding sites on HRF were mapped, one to the N-terminus (residues 1–19) and another to a helical region (residues 107–135) (Kashiwakura et al., 2012). HRF can form dimers (Yoon et al., 2000; Kim et al., 2009, Kashiwakura et al., 2012), and a disulphide-linked dimer was shown to be crucial for the cytokine-like activity of rat HRF (Kim et al., 2009). mHRF, hHRF and rat HRF contain two cysteine residues, at positions 28 and 172, and Cys172 is suggested to be the site of the intermolecular disulphide bond (Kim et al., 2009).

The current model for the activity of HRF in mast cell activation involves cross-linking of FcεRI-bound IgE by dimeric HRF, mediated by interactions between HRF and the Fab regions of IgE (Kawakami et al., 2014). HRF crystal structures have revealed different packing arrangements, but provided little insight into the formation of dimers, particularly those linked by a disulphide bond. Crystal structures for HRF from *Plasmodium knowlesi* (Vedadi et al., 2007) and *Plasmodium falciparum* (Eichhorn et al., 2013) contain monomers in their asymmetric units; both proteins contain a cysteine residue which is buried and thus incapable of forming a disulphide-linked dimer. Likewise, the solution structure of HRF from *Schizosaccharomyces pombe* (Thaw et al., 2001) comprises a monomer, and the side chain of the single cysteine residue is buried. Although HRF from *Caenorhabditis elegans* contains two cysteine residues which are surface exposed, the solution structure also comprises a monomer (Lange et al., 2012). The solution structure of hHRF (Feng et al., 2007) also reveals a monomeric structure, and one crystal structure contains four molecules in the asymmetric unit (Susini et al., 2008) which form only non-covalent interactions with one another. To date, a single crystal structure of an hHRF Glu12Val mutant has revealed a disulphide-linked dimer, mediated by Cys172 (Dong et al., 2009), but the two monomers are not closely associated with one another, and the C-terminal purification tag of the construct contributes a substantial portion of the dimer interface.

We report here the first crystal structure of murine HRF (mHRF), solved at 4.0 Å resolution, revealing the conserved HRF fold. We also report two structures of human HRF (hHRF) in new crystal forms, one of which was solved at the highest resolution yet reported (1.4 Å) for HRF. One hHRF structure, and the mHRF structure, contain non-covalent HRF interactions, but reveal different packing arrangements. However, the high resolution hHRF structure reveals a disulphide-linked HRF dimer, with substantial contact between the two monomers, finally providing a model for the activity of dimeric HRF in allergic disease.

## 2 Materials and methods

### 2.1 Protein preparation and crystallisation

mHRF and hHRF were prepared according to a previously described protocol (Kashiwakura et al., 2012). Both proteins include a C-terminal His-tag for purification. mHRF crystals were grown at 18 °C using the sitting drop vapour diffusion method, with a reservoir volume of 70 μL and drops comprising 100 nL protein (4.7 mg/mL) and 50 nL reservoir. mHRF crystals were grown in 0.1 M Tris-HCl pH8.4, 23% (w/v) PEG 2000 MME and 0.01 M nickel chloride, and were cryoprotected in 0.1 M Tris-HCl pH8.4, 23% (w/v) PEG 2000 MME, 0.01 M nickel chloride and 20% (v/v) glycerol. hHRF crystals were grown at 18 °C using the sitting drop vapour diffusion method, with a reservoir volume of 50 μL and drops comprising 120 nL protein (10 mg/mL) and 120 nL reservoir. hHRF-1 crystals were grown in 0.1 M MMT pH4.0 and 25% (w/v) PEG 1500, and were cryoprotected using the mother liquor. hHRF-2 crystals were grown in 0.1 M MES pH6.0 and 20% (w/v) PEG 2000 MME and were also cryoprotected using the mother liquor.

### 2.2 Structure determination, model building and refinement

Data were collected at beamlines I04 (mHRF), I03 (hHRF-1) and I04-1 (hHRF-2) at the Diamond Light Source (Harwell, UK). Data were integrated with XDS (Kabsch, 2010) or DIALS (Waterman et al., 2013) within the *xia2* program (Winter, 2010), and scaled with AIMLESS (Evans and Murshudov, 2013) from the CCP4 suite (Winn et al., 2011). The mHRF crystals diffracted anisotropically, and the data were truncated to resolution limits of 4.2 Å, 4.5 Å and 4.0 Å using the UCLA Diffraction Anisotropy Server (Strong et al., 2006). mHRF and hHRF structures were solved by molecular replacement with PHASER (McCoy et al., 2007) using PDB 1YZ1 (Susini et al., 2008) as a search model. For all structures, refinement was performed with PHENIX (Adams et al., 2010) and manual model building with *Coot* (Emsley et al., 2010). Data processing and refinement statistics are presented in Table 1. Interfaces were analysed with PISA (Krissinel and Henrick, 2007) and figures were prepared with PyMOL (Version 1.8.2.1 Schrödinger, LLC).

**Table 1:** Data processing and refinement statistics.

Data processing	mHRF	hHRF-1	hHRF-2
Space group	P 2_1_ 2_1_ 2	P 1 2_1_ 1	P 2_1_ 2_1_ 2_1_
Unit cell dimensions (Å)	*a* = 69.53	*a* = 49.73	*a* = 47.53
	*b* = 99.85	**b* = *58.95	*b* = 77.59
	*c* = 57.47	**c* = *57.73	*c* = 99.32
		β* = *99*.*41°	
Resolution (Å)			
Overall	57.06−4.01	24.53−1.75	19.86−1.40
(outer shell)	(4.63−4.01)	(1.78−1.75)	(1.43−1.40)
Completeness (%)^a^	98.5 (95.8)	99.3 (97.3)	99.7 (99.6)
Multiplicity^a^	11.6 (12.0)	4.5 (3.4)	7.4 (7.6)
Mean ((I)/σ(I))^a^	9.0 (2.1)	11.7 (1.8)	12.7 (1.9)
R_merge_^a^	0.271 (1.892)	0.124 (1.085)	0.066 (1.112)
R_pim_^a^	0.081 (0.554)	0.065 (0.667)	0.025 (0.428)
CC_1/2_^a^	0.997 (0.877)	0.988 (0.342)	0.999 (0.703)
Wilson *B*-factor (Å^2^)	233	15.9	15.1

Refinement			
R_work_/R_free_ (%)^b^	29.96/34.16	18.84/22.09	17.93/19.85
No. of reflections	3 125^c^	32 846	72 706
RMSD			
Bond lengths (Å)	0.001	0.004	0.015
Bond angles (°)	0.361	0.632	1.546
Coordinate error (Å)	0.48	0.20	0.14
No. of atoms			
Protein	2 159	2 564	2 581
Solvent	0	215	301
Other	6^d^	0	5^e^
Ramachandran plot			
Favoured (%)	97.12	99.05	98.48
Allowed (%)	100	100	100

a Values in parentheses are for the highest resolution shell.

b R_free_ set comprises 5% of reflections.

c Data were truncated to resolution limits of 4.2 Å, 4.5 Å and 4.0 Å.

d Glycerol.

e Polyethylene glycol.

## 3 Results and discussion

We report here the first crystal structure of mHRF at 4.0 Å resolution, and two structures of hHRF (hHRF-1 and hHRF-2) in new crystal forms, the latter revealing a disulphide-linked dimer.

### 3.1 Overall structure of mHRF

**Fig. 1:**
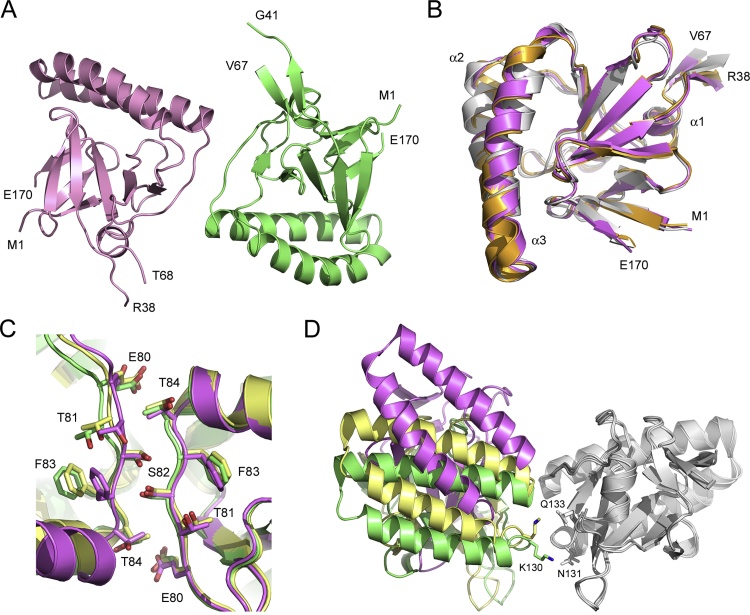
mHRF. (A) Overall structure of mHRF solved at 4.0 Å resolution. The two molecules of the asymmetric unit are coloured pink and green, and the positions of the N- and C-terminal residues, and residues adjacent to the disordered loop, are indicated. (B) The overall fold of mHRF is conserved. The solution structure (grey) (Feng et al., 2007) and a crystal structure (orange) (Susini et al., 2008) of human HRF are superposed on chain A from the mHRF crystal structure (purple). The three α-helices are labeled, and the positions of the N- and C-terminal residues, and residues adjacent to the disordered loop, are indicated. For clarity, the mobile loop from the solution structure is not shown. (C) Interface between Glu80-Thr84 in molecules related by non-crystallographic two-fold symmetry. mHRF is coloured purple, and hHRF structures are coloured green [PDB 1YZ1 (Susini et al., 2008)] and yellow [PDB 3EBM (Dong et al., 2009)]. (D) The overall positions of the two molecules differ in the mHRF and hHRF crystal structures. mHRF and hHRF structures were superposed on one of the two molecules related by non-crystallographic two-fold symmetry (grey), and the second molecule is coloured as follows: mHRF (purple), PDB 1YZ1 (green) and PDB 3EBM (yellow). Lys130 from one chain, and Asn131 and Gln133 from the other are labeled for structures 1YZ1 and 3EBM. (For interpretation of the references to colour in this figure legend, the reader is referred to the web version of this article.)

The structure of mHRF was solved at 4.0 Å resolution, and contains two molecules in the asymmetric unit (Fig. 1A). Residues 1–38 and 68–170, and 1–41 and 67–170 were modeled for chains A and B, respectively; similar to other crystal structures of HRF (Vedadi et al., 2007, Susini et al., 2008, Dong et al., 2009, Eichhorn et al., 2013), the mobile loop region (residues Thr39-Val66) was disordered. mHRF contains a C-terminal His-tag, which was also disordered; a substantial region of continuous electron density was observed close to the C-terminus, but the tag could not be modeled with certainty.

The overall fold of mHRF is conserved, and comprises three α-helices packed against two β-sheets, while a third β-sheet forms the base of the mobile loop (Fig. 1B). Consistent with a conserved fold, the individual monomers of the mHRF asymmetric unit were superposed on HRF crystal structures (*H. sapiens*, *P. falciparum* and *P. knowlesi*) (Vedadi et al., 2007, Susini et al., 2008, Dong et al., 2009, Eichhorn et al., 2013) with RMSD values for Cα atoms ranging from 0.52-1.22 Å, and on HRF solution structures (*C. elegans, H. sapiens* and *S. pombe*) (Thaw et al., 2001, Feng et al., 2007, Lange et al., 2012) with RMSD values ranging from 1.16-3.23 Å.

Like hHRF, mHRF contains two cysteine residues at positions 28 and 172, and thus has the potential to form a covalently-linked dimer; while Cys28 is buried, Cys172 is surface exposed. However, the two molecules of the mHRF asymmetric unit, which are related by non-crystallographic two-fold symmetry, form only non-covalent interactions (Fig. 1A). The two molecules bury a surface area of 176 Å^2^ at their interface, in which Glu80-Thr84 from one chain pack against equivalent residues from the other, with a hydrogen bond between Ser82 from each chain (Fig. 1C). A similar interface is found in two previously reported hHRF structures [PDB 1YZ1 (Susini et al., 2008) and PDB 3EBM (Dong et al., 2009)]; however, the molecules in these two structures are more closely associated with one another (Fig. 1D), and the interface buries a surface area of up to 346 Å^2^. Lys130 plays a key role at this interface: Lys130 from one chain contacts Lys130, Asn131 or Gln133 from the other. By contrast, these interactions are precluded in mHRF, as not only do the overall positions of the two molecules differ, but residue 130 is one of the seven positions at which the mHRF and hHRF sequences diverge (Asn130 in mHRF/Lys130 in hHRF).

### 3.2 Overall structure of hHRF-1

**Fig. 2:**
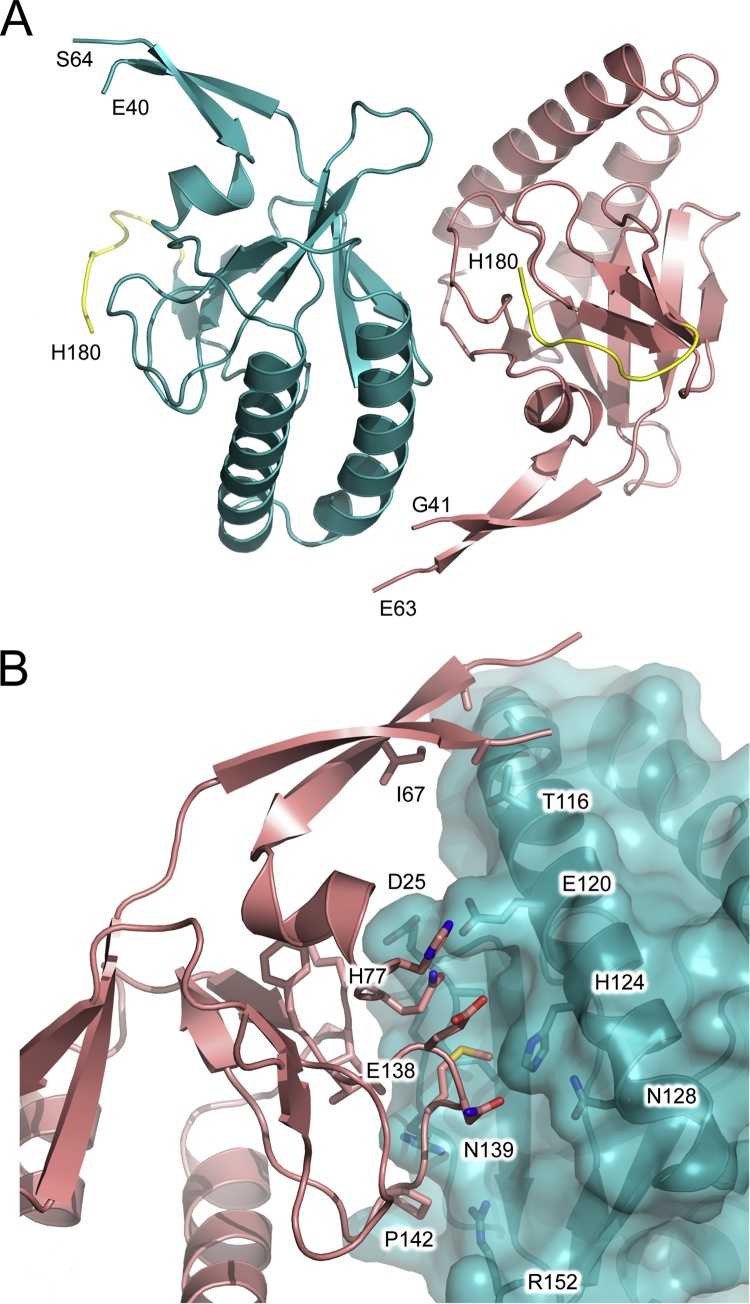
hHRF-1. (A) Overall structure of hHRF-1 solved at 1.75 Å resolution. The two molecules of the asymmetric unit are coloured blue and salmon. The C-terminal tag is coloured yellow and the positions of C-terminal residues, and residues adjacent to the disordered loop, are indicated. (B) The two monomers of the asymmetric unit (blue and salmon) bury a surface area of ∼530 Å^2^ at their interface. (For interpretation of the references to colour in this figure legend, the reader is referred to the web version of this article.)

The structure of hHRF-1, solved at 1.75 Å resolution, contains two molecules in the asymmetric unit (Fig. 2A). Residues 1–41 and 62–180, and 1–40 and 64–180 were modeled for chains A and B of the hHRF-1 structure, respectively, and as in the mHRF structure, a substantial portion of the mobile loop region was disordered.

The two molecules of the asymmetric unit form non-covalent interactions, and are arranged such that their interface buries a surface area of ∼530 Å^2^ (Fig. 2B). This particular packing arrangement is very different to that of mHRF, and has not been observed in any other HRF crystal structure. The interface includes the following interactions: Glu40, Thr65 and Ile67 (chain A) pack against Thr116 (chain B); Gln79 (chain A) forms a hydrogen bond with Glu120 (chain B), and together with Glu80 (chain A) contacts Asp25 (chain B), which also forms a hydrogen bond with the Gln80 (chain A) main chain; Ser82 (chain A) contacts Glu22 (chain B), and a water molecule forms a bridging hydrogen bond with the Glu22 main chain; Asn139 (chain A) contacts His124 (chain B), and a water molecule forms a bridging hydrogen bond between the Asn139 main chain and His124 side chain; Met140 (chain A) contacts Ile23, Ala24, Gln121 and His124 (chain B), and the Met140 main chain forms a hydrogen bond with Arg21 (chain B); Pro142 (chain A) packs against Arg21 and Arg152 (chain B).

### 3.3 Overall structure of hHRF-2

**Fig. 3:**
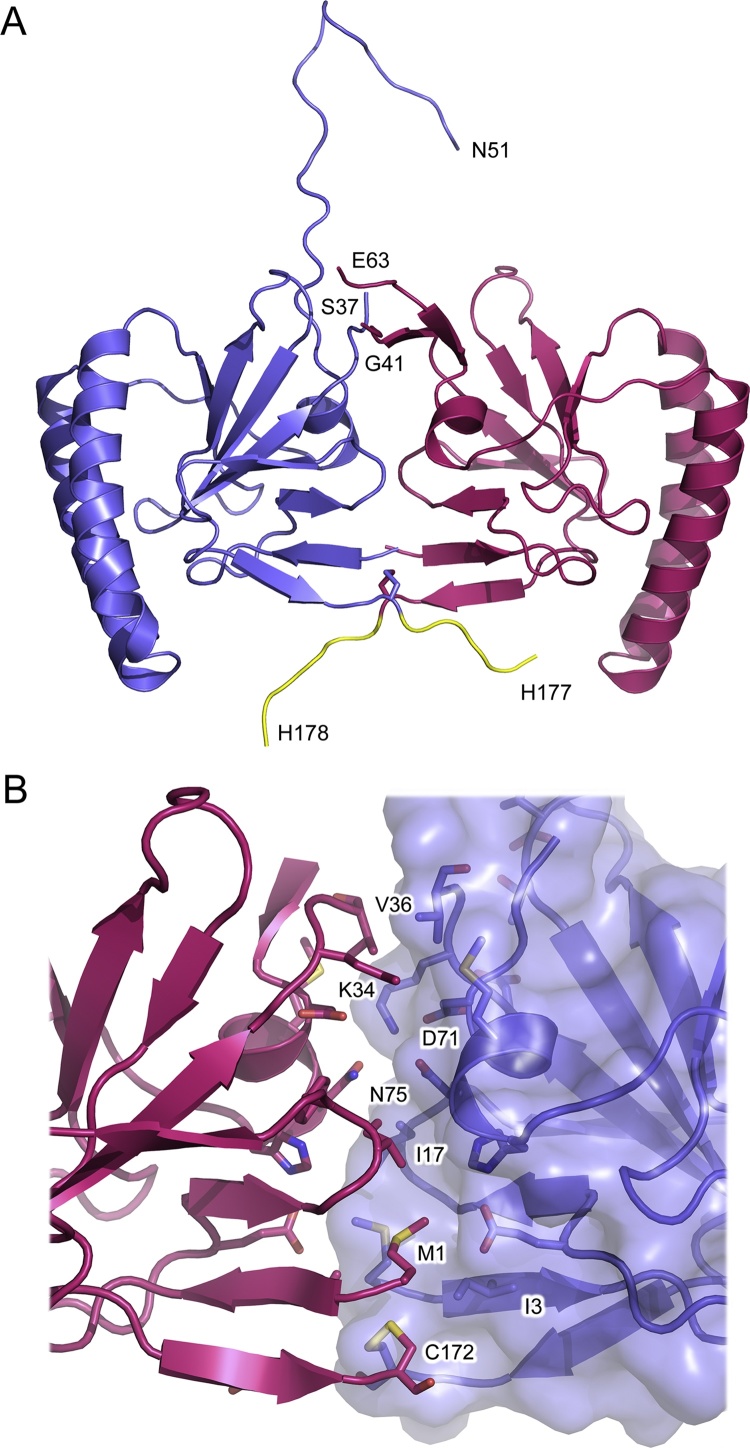
hHRF-2. (A) Overall structure of hHRF-2, solved at 1.4 Å resolution. The two molecules of the asymmetric unit are coloured blue and pink. For each structure, the C-terminal tag is coloured yellow and the positions of C-terminal residues, and residues adjacent to the disordered loop, are indicated. (B) The two monomers of the hHRF dimer (blue and pink) bury a surface area of ∼954 Å^2^ at their interface (∼666 Å^2^ if the C-terminal His-tag is omitted from the structure). (For interpretation of the references to colour in this figure legend, the reader is referred to the web version of this article.)

The structure of hHRF-2 was solved at 1.4 Å resolution, the highest reported to date for HRF, and contains two molecules in the asymmetric unit (Fig. 3A). Residues 1–41 and 63–178, and 1–37 and 50–177 were modeled for chains A and B of the hHRF-2 structure, respectively. There is an extensive dimer interface of ∼954 Å^2^, which will be discussed in detail in the following section.

**Fig. 4:**
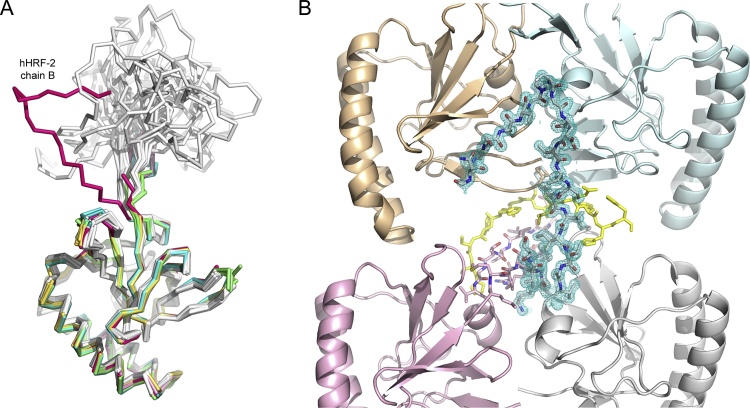
The hHRF mobile loop. (A) In chain B of the hHRF-2 structure, the mobile loop conformation differs from that in the hHRF solution structure, and partially ordered loops in other hHRF crystal structures. Structures are coloured as follows: hHRF NMR structure (PDB 2HR9, 20 conformers), grey (Feng et al., 2007); hHRF crystal structure (PDB 1YZ1, four chains), green (Susini et al., 2008); hHRF crystal structure (PDB 3EBM, four chains), yellow (Dong et al., 2009); hHRF-1 structure chain A, pale blue; hHRF-1 structure chain B. teal; hHRF-2 structure chain A, pale pink; hHRF-2 structure chain B, dark pink. For clarity, C-terminal tags are not shown. (B) The mobile loop from chain B of the hHRF-2 structure packs against a symmetry-related dimer. Chains A and B of the hHRF-2 structure are coloured pink and grey, respectively, and loop residues are shown as sticks. Electron density is shown for the loop from chain B (2F_o_F_c_ map contoured at 1σ). Symmetry-related molecules are coloured in pale orange and blue, and the C-terminal tag is coloured yellow. (For interpretation of the references to colour in this figure legend, the reader is referred to the web version of this article.)

In HRF crystal and solution structures, the base of the mobile loop consists of two strands of β-sheet, and the overall position of this sheet is conserved. In chain A of the hHRF-2 structure, residues Asn42-Thr62 of the mobile loop are disordered, similar to that seen in mHRF. By contrast, only residues Arg38-Gly49 are disordered in chain B; this chain contains the greatest number of mobile loop residues modeled in any HRF crystal structure to date. However, in chain B, the ordered base of the mobile loop does not form a β-sheet, and the conformation of the main chain differs from other hHRF structures (Fig. 4A). The overall position of the ordered part of the mobile loop in this chain also differs from those observed in the hHRF solution structure (Feng et al., 2007), and is stabilized by packing interactions with symmetry-related molecules (Fig. 4B). These interactions include: a hydrogen bond between the Ala52 main chain and Glu22 side chain (from chain B of a symmetry-related molecule), two hydrogen bonds between the Ala54 main chain and Ile20 main chain (from chain B of a symmetry-related molecule), packing of Gly61 against His77, with a hydrogen bond between the Gly61 main chain and Glu138 (from chain A of a symmetry-related molecule), and packing of Thr65 against the C-terminal His-tag (from chain B of a symmetry-related molecule).

The different mobile loop conformations observed in the monomeric hHRF solution structure (Feng et al., 2007) are incompatible with the hHRF-2 crystal structure lattice due to clashes with symmetry-related molecules, or the other molecule of the asymmetric unit. It is unclear whether the conformation of the partially ordered mobile loop in chain B of the hHRF-2 structure would be adopted in solution, but this structure adds to our understanding of the flexibility of the mobile loop by defining a new, accessible conformation.

### 3.4 hHRF-2 contains a disulphide-linked dimer

**Fig. 5:**
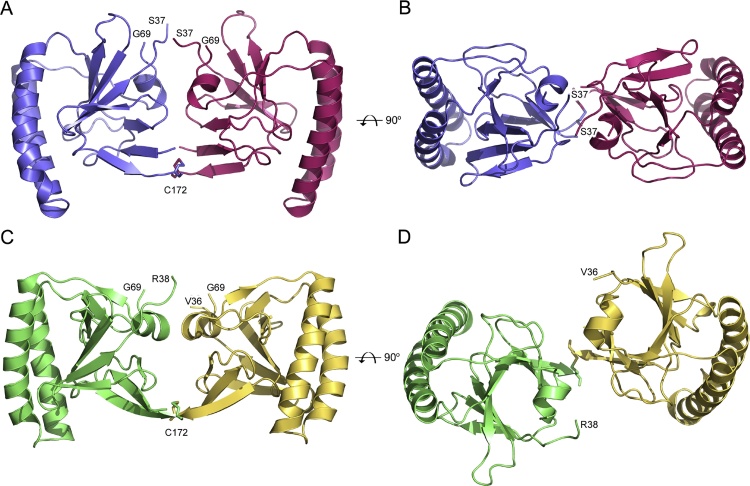
Disulphide-linked hHRF dimers. (A) Disulphide-linked dimer of hHRF-2. The two monomers are coloured blue and pink. Residues 50–68 from the mobile loop in chain B (blue) have been omitted for clarity. (B) View of the hHRF-2 dimer after a 90° rotation towards the reader. (C) Disulphide-linked dimer from PDB 3EBM (Dong et al., 2009). The two monomers are coloured green and yellow. (D) View of the 3EBM dimer after a 90° rotation towards the reader. The position of Cys172, involved in inter-chain disulphide bond formation, is indicated for each structure. (For interpretation of the references to colour in this figure legend, the reader is referred to the web version of this article.)

hHRF contains two cysteine residues, at positions 28 and 172, and Cys172 is suggested to be responsible for disulphide bond-mediated dimerisation (Kim et al., 2009); notably, Cys172 is surface exposed, while Cys28 is buried, in the structure of the hHRF monomer. In the hHRF-2 structure, the two molecules of the asymmetric unit are related to one another by non-crystallographic two-fold symmetry, and contain an intermolecular disulphide bond between Cys172 from each chain (Figs. 5A and 5B).

The interface between the two molecules includes the following contacts: the base of the two mobile loops (residues 34–39 in chain A and 33–37 in chain B) pack against one another; Met1 from one chain contacts Met1, Ile3 and Glu12 from the other; Ile17 packs against Asn75 and His76, while Met74 packs against the mobile loop; furthermore, a single water molecule forms two hydrogen bonds, one with Asn75 in each chain. The disulphide bond between Cys172 in each chain is accommodated in a shallow depression created by the Met1 and Ile3 side chains (Fig. 3B). Due to the two-fold symmetrical nature of the interaction, the interface is similar for each chain, but not identical due to the different conformation of the mobile loop in chain B.

Only one other HRF structure [human HRF containing a Glu12Val mutation (PDB 3EBM)], contains a dimer covalently linked by a disulphide bond (Dong et al., 2009). In this structure, in which the two monomers are also related by non-crystallographic two-fold symmetry (Figs. 5C and D), Met1 contacts Glu170 from the other chain, residues His77 from each chain pack against one another, and additionally form a hydrogen bond with Glu138 from the other chain. The disulphide bond is more exposed than in the hHRF-2 structure, and packs against Met1, Ile3 and Glu170. Notably, Glu12 is found at the dimer interface in wild-type HRF (hHRF-2 structure), its side chain forming a water-mediated bridging hydrogen bond with the second monomer. By contrast, the two monomers are arranged differently in the Glu12Val mutant dimer, and Val12 would be unable to form a comparable hydrogen bond.

Wild-type hHRF described here, and the Glu12Val hHRF mutant, both contain a C-terminal purification tag (Leu173-His180), and in each dimer the tag in one chain contacts the other monomer; however, this interaction differs between the two structures. In the structure of the Glu12Val hHRF mutant (Dong et al., 2009), the Leu173 main chain forms hydrogen bonds with the Lys171 main chain, while His175 packs against Ile2, Met169 and Lys171 from the other monomer. By contrast, in the wild-type structure hHRF-2, a water molecule forms bridging hydrogen bonds between Leu173 main chain atoms, while His175 packs against Glu170. In chain B of the hHRF-2 structure, Glu174 packs against Arg5, while His177 packs against His10 (Glu174 is partially disordered in chain A, and in each chain the overall conformation of the tag differs after His176).

The interface between the two molecules of the Glu12Val hHRF mutant buries a surface area of ∼652 Å^2^, of which 40% is contributed by the C-terminal His-tag. By contrast, the interface in hHRF-2 buries a substantially larger surface area, ∼954 Å^2^, of which a smaller proportion, ∼25%, is contributed by the tag. Thus, the two molecules in the wild-type hHRF-2 dimer are much more closely associated with one another, compared with the mutant (Figs. 5A-D), and we propose that this new structure reflects the native dimeric and functionally active state with respect to mast cell activation.

### 3.5 hHRF-2 structure reveals a model for the role of HRF in mast cell activation

The current model for the activity of HRF in mast cell activation is that dimeric HRF cross-links FcεRI-bound IgE on the mast cell surface, mediated by interactions between HRF and the Fab regions of a subset of IgE molecules (Kawakami et al., 2014). IgE binding sites on HRF, which interact with the Fabs, have been mapped to the N-terminal region (residues 1–19) and helix 3 (residues 107–135) (Kashiwakura et al., 2012). HRF can form a disulphide-linked dimer, mediated by Cys172 (Kim et al., 2009), and the dimer is able to bind to IgE (Kashiwakura et al., 2012).

**Fig. 6:**
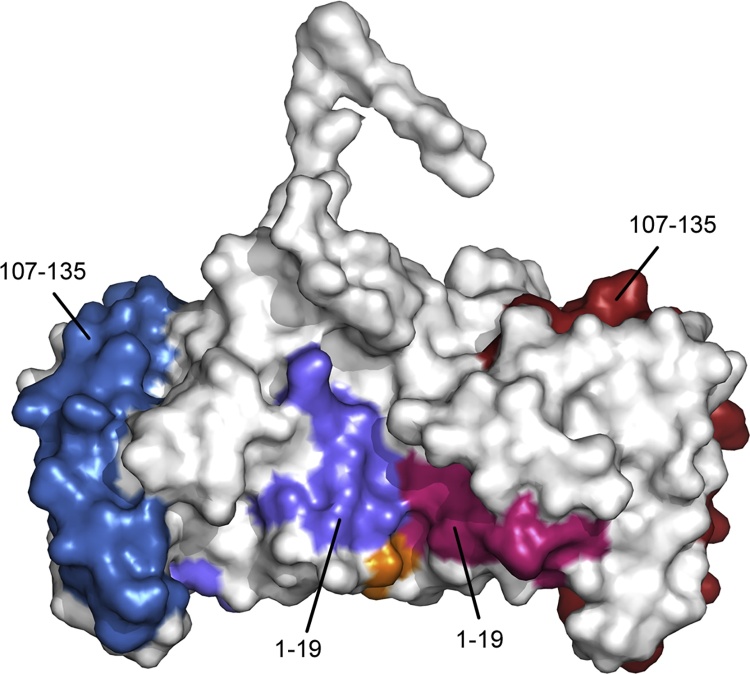
The hHRF-2 disulphide-linked dimer is consistent with a model of mast cell activation. The two monomers of the hHRF-2 dimer are coloured white and Cys172 is coloured orange. For the first monomer, the two IgE binding sites, mapped to residues Met1-Lys19, and Arg (hHRF)/Lys (mHRF) 107-Ile135, are coloured light blue and dark blue respectively. For the second monomer, residues 1–19 and 107–135, are coloured light pink and dark pink respectively. This dimeric structure offers a model not only for human, but also murine, HRF in mast cell activation. (For interpretation of the references to colour in this figure legend, the reader is referred to the web version of this article.)

The hHRF-2 structure comprises a wild-type hHRF dimer, with a disulphide bond between Cys172 from each monomer. In this dimer, the two monomers are closely associated, burying a surface area of ∼666 Å^2^ if the C-terminal His-tag is omitted from the structure. Crucially, the two binding sites necessary for the Fab interactions are surface exposed in each molecule (Fig. 6), and would thus enable cross-linking of FcεRI-bound IgE to occur. Furthermore, the human and murine proteins are 96% identical, and the residues involved in forming both the disulphide bond and dimer interface are identical. We therefore propose that the hHRF-2 structure provides a model for the activity of both human and murine HRF in mast cell activation.

## 4 Conclusions

We report the first crystal structure of murine HRF and the structure of human HRF in two new crystal forms, one at the highest resolution yet reported. The high resolution human HRF structure contains a disulphide linked dimer, with closely packed monomers, which provides a model for the activity of human and murine HRF in allergic disease.

## Accession numbers

Coordinates and structure factors have been deposited in the Protein Data Bank with accession numbers: mHRF, PDB: 5O9K; hHRF-1 PDB 5O9L; hHRF-2, PDB 5O9M.

## References

[bib0005] Adams P.D., Afonine P.V., Bunkóczi G., Chen V.B., Davis I.W., Echols N., Headd J.J., Hung L.-W., Kapral G.J., Grosse-Kunstleve R.W., McCoy A.J., Moriarty N.W., Oeffner R., Read R.J., Richardson D.C., Richardson J.S., Terwilliger T.C., Zwart P.H. (2010). PHENIX: a comprehensive Python-based system for macromolecular structure solution. Acta Crystallogr. D Biol. Crystallogr..

[bib0010] Bommer U.A., Thiele B.J. (2004). The translationally controlled tumour protein (TCTP). Int. J. Biochem. Cell Biol..

[bib0015] Bommer U.A. (2012). Cellular function and regulation of the translationally controlled tumour protein TCTP. Open Allergy J..

[bib0020] Dong X., Yang B., Li Y., Zhong C., Ding J. (2009). Molecular basis of the acceleration of the GDP-GTP exchange of human Ras homolog enriched in brain by human Translationally Controlled Tumor Protein. J. Biol. Chem..

[bib0025] Eichhorn T., Winter D., Büchele B., Dirdjaja N., Frank M., Lehmann W.D., Mertens R., Krauth-Siegel R.L., Simmet T., Granzin J., Efferth T. (2013). Molecular interaction of artemisinin with translationally controlled tumor protein (TCTP) of *Plasmodium falciparum*. Biochem. Pharmacol..

[bib0030] Emsley P., Lohkamp B., Scott W.G., Cowtan K. (2010). Features and development of *Coot*. Acta Crystallogr. D Biol. Crystallogr..

[bib0035] Evans P.R., Murshudov G.N. (2013). How good are my data and what is the resolution?. Acta Crystallogr. D Biol. Crystallogr..

[bib0040] Feng Y., Liu D., Yao H., Wang J. (2007). Solution structure and mapping of a very weak calcium-binding site of human translationally controlled tumor protein by NMR. Arch. Biochem. Biophys..

[bib0045] Gould H.J., Sutton B.J. (2008). IgE in allergy and asthma today. Nat. Rev. Immunol..

[bib0050] Kabsch W. (2010). XDS. Acta Crystallogr. D Biol. Crystallogr..

[bib0055] Kashiwakura J.C., Ando T., Matsumoto K., Kimura M., Kitaura J., Matho M.H., Zajonc D.M., Ozeki T., Ra C., MacDonald S.M., Siraganian R.P., Broide D.H., Kawakami Y., Kawakami T. (2012). Histamine-releasing factor has a proinflammatory role in mouse models of asthma and allergy. J. Clin. Invest..

[bib0060] Kawakami T., Kashiwakura J., Kawakami Y. (2014). Histamine-Releasing factor and immunoglobulins in asthma and allergy. Allergy Asthma Immunol. Res..

[bib0065] Kim M., Min H.J., Won H.Y., Park H., Lee J.-C., Park H.-W., Chung J., Hwang E.S., Lee K. (2009). Dimerization of translationally controlled tumor protein is essential for its cytokine-like activity. PLoS One.

[bib0070] Krissinel E., Henrick K. (2007). Inference of macromolecular assemblies from crystalline state. J. Mol. Biol..

[bib0075] Lange O.F., Rossi P., Sgourakis N.G., Song Y., Lee H.W., Aramini J.M., Ertekin A., Xiao R., Acton T.B., Montelione G.T., Baker D. (2012). Determination of solution structures of proteins up to 40 kDa using CS-Rosetta with sparse NMR data from deuterated samples. Proc. Natl. Acad. Sci. USA..

[bib0080] MacDonald S.M., Rafnar T., Langdon J., Lichtenstein L.M. (1995). Molecular identification of an IgE-dependent histamine-releasing factor. Science.

[bib0085] McCoy A.J., Grosse-Kunstleve R.W., Adams P.D., Winn M.D., Storoni L.C., Read R.J. (2007). Phaser crystallographic software. J. Appl. Crystallogr..

[bib0090] Nagano-Ito M., Ichikawa S. (2012). Biological effects of mammalian translationally controlled tumor protein (TCTP) on cell death, proliferation, and tumorigenesis. Biochem. Res. Int..

[bib0095] Strong M., Sawaya M.R., Wang S., Phillips M., Cascio D., Eisenberg D. (2006). Toward the structural genomics of complexes: crystal structure of a PE/PPE protein complex from *Mycobacterium tuberculosis*. Proc. Natl. Acad. Sci. USA..

[bib0100] Susini L., Besse S., Duflaut D., Lespagnol A., Beekman C., Fiucci G., Atkinson A.R., Busso D., Poussin P., Marine J.C., Martinou J.C., Cavarelli J., Moras D., Amson R., Telerman A. (2008). TCTP protects from apoptotic cell death by antagonizing bax function. Cell Death Differ..

[bib0105] Thaw P., Baxter N.J., Hounslow A.M., Price C., Waltho J.P., Craven C.J. (2001). Structure of TCTP reveals unexpected relationship with guanine nucleotide-free chaperones. Nat. Struct. Biol..

[bib0110] Vedadi M., Lew J., Artz J., Amani M., Zhao Y., Dong A., Wasney G.A., Gao M., Hills T., Brokx S., Qiu W., Sharma S., Diassiti A., Alam Z., Melone M., Mulichak A., Wernimont A., Bray J., Loppnau P., Plotnikova O., Newberry K., Sundararajan E., Houston S., Walker J., Tempel W., Bochkarev A., Kozieradzki I., Edwards A., Arrowsmith C., Roos D., Kain K., Hui R. (2007). Genome-scale protein expression and structural biology of *Plasmodium falciparum* and related Apicomplexan organisms. Mol. Biochem. Parasitol..

[bib0115] Waterman D.G., Winter G., Parkhurst J.M., Fuentes-Montero L., Hattne J., Brewster A., Sauter N.K., Evans G. (2013). The DIALS framework for integration software. CCP4 Newsl. Protein Crystallogr..

[bib0120] Winn M.D., Ballard C.C., Cowtan K.D., Dodson E.J., Emsley P., Evans P.R., Keegan R.M., Krissinel E.B., Leslie A.G.W., McCoy A., McNicholas S.J., Murshudov G.N., Pannu N.S., Potterton E.A., Powell H.R., Read R.J., Vagin A., Wilson K.S. (2011). Overview of the CCP4 suite and current developments. Acta Crystallogr. D Biol. Crystallogr..

[bib0125] Winter G. (2010). *xia2*: an expert system for macromolecular crystallography data reduction. J. Appl. Crystallogr..

[bib0130] Yoon T., Jung J., Kim M., Lee K.M., Choi E.C., Lee K. (2000). Identification of the self-interaction of rat TCTP/IgE-dependent histamine-releasing factor using yeast two-hybrid system. Arch. Biochem. Biophys..

